# Implication of intracellular chloride channel in extracellular matrix remodeling in pressure‐overloaded mice and patients with dilated cardiomyopathy

**DOI:** 10.14814/phy2.70726

**Published:** 2026-01-11

**Authors:** Gaku Oguri, Seitaro Nomura, Takafumi Nakajima, Hironobu Kikuchi, Syotaro Obi, Issei Komuro, Norihiko Takeda, Shigeru Toyoda, Toshiaki Nakajima

**Affiliations:** ^1^ Department of Cardiovascular Medicine The University of Tokyo Tokyo Japan; ^2^ Department of Frontier Cardiovascular Science The University of Tokyo Tokyo Japan; ^3^ Department of Cardiovascular Medicine Dokkyo Medical University and Heart Center, Dokkyo Medical University Hospital Tochigi Japan

**Keywords:** chloride intracellular channels, extracellular matrix remodeling, heart failure, hypertrophy

## Abstract

Chloride intracellular channels (CLICs) are important in cardiac cellular physiology. We aimed to determine the pathophysiological roles of CLICs in the heart. For this, we analyzed *CLIC* expression in cardiomyocytes in a mouse transverse aortic constriction (TAC) model to induce cardiac hypertrophy and failure, as well as in ventricular myocytes from patients with dilated cardiomyopathy (DCM) using single‐cell RNA‐sequencing. Single‐ventricular myocytes were isolated from the left ventricular free wall of C57BL/6J mice after TAC (pre‐TAC; Day 3 post‐TAC; and Weeks 1, 2, 4, and 8 post‐TAC). Gene expression was compared with data from sham controls. In mice, *CLIC1* and *CLIC4* expression significantly increased in Day 3 and Weeks 1, 2, and 4 post‐TAC. *CLIC5* expression showed an increase during all phases. Kyoto Encyclopedia of Genes and Genomes pathway analysis for genes associated with *CLIC1*, *CLIC4*, and *CLIC5* revealed a strong association between focal adhesion activation and actin cytoskeleton regulation pathways linked to extracellular matrix (ECM) remodeling. *CLIC1* and *CLIC4* expression was also higher in cells from patients with DCM. Single‐cell RNA‐sequencing revealed the possible role of CLICs in myocardial ventricular remodeling linked to ECM, proposing their potential as therapeutic targets for cardiac hypertrophy and failure.

## 1 INTRODUCTION

Cardiac hypertrophy is an adaptive response to increased cardiac workload that occurs in both physiological and pathological contexts (Shimizu & Minamino, 2016). The development of cardiac hypertrophy involves complex mechanisms, including mechanotransduction, angiogenesis, and metabolic changes, which contribute to cardiac remodeling and dysfunction (Lyon et al., 2015; Oka et al., 2014). Further, pathological hypertrophy is characterized by fibrosis, capillary rarefaction, and cellular dysfunction, ultimately resulting in cardiac remodeling and functional decline (Shimizu & Minamino, 2016) and heart failure progression (Dorn, 2007). Several studies using proteomic analysis of heart failure have revealed changes in the expression of proteins involved in cardiac energy metabolism, mitochondrial dysfunction, and the cardiac extracellular matrix (ECM) (Lu et al., 2019; Roselló et al., 2012). Understanding the mechanisms underlying cardiac hypertrophy and heart failure is crucial for the development of novel therapeutic strategies to prevent or reverse its detrimental effects (Frey & Olson, 2003).

The ECM network plays a crucial role in cardiac homeostasis by providing structural support, facilitating force transmission, and transducing key signals to cardiomyocytes, vascular cells, and interstitial cells. Its main components are proteins such as collagen and elastin, which play a role in maintaining the heart's elasticity and strength. Changes in the profile and biochemistry of the ECM may be critically implicated in the pathogenesis of hypertrophy and heart failure. Ion channels and ECM components engage in reciprocal regulatory interactions. ECM remodeling enzymes, such as matrix metalloproteinases (MMPs), and mechanical cues induced by matrix stiffening modulate ion channel activity by altering the biochemical microenvironment and releasing bioactive factors that influence channel function (Bonnans et al., 2014; Lee et al., 2024; Zhang et al., 2025). Conversely, ion channels regulate ECM integrity by controlling intracellular calcium signaling, pH homeostasis, and the secretion of proteolytic enzymes, thereby influencing collagen deposition, fibronectin assembly, and the overall ECM architecture (Dzobo & Dandara, 2023; Ji & Mcculloch, 2021). In addition, ion channels, particularly mechanosensitive channels such as TRPM7, are key modulators of cardiac fibroblast function, including proliferation, differentiation, and ECM turnover (Stewart & Turner, 2021; Yue et al., 2013). Thus, understanding the interplay between ion channels and ECM components is crucial for developing new therapeutic strategies to manage fibrosis and promote physiological ECM repair in heart disease (Silva et al., 2020). Chloride channels are ubiquitously expressed, localized both in the plasma membrane and in intracellular organelles, and are involved in ion homeostasis, cell volume regulation, transepithelial transport, and regulation of electrical excitability (Jentsch et al., 2002). Among them, chloride intracellular channels (CLICs) are non‐canonical ion channels with six homologs in mammals, existing as either soluble or integral membrane protein forms, with dual functions as enzymes and channels (Gururaja Rao et al., 2018). CLIC4 is enriched in the outer mitochondrial membrane, and CLIC5 is present in the inner mitochondrial membrane, with directly modulating mitochondrial reactive oxygen species (ROS) generation (Gururaja Rao et al., 2020; Ponnalagu et al., 2016). Previous studies have reported the involvement of CLICs (CLIC1, CLIC4, CLIC5) in atrial fibrosis and remodeling in atrial fibrillation (AF) (Jiang et al., 2017). In pulmonary arterial hypertension, CLIC4 gene deletion markedly attenuated the development of chronic hypoxia‐induced pulmonary hypertension in mice (Wojciak‐Stothard et al., 2014). These results suggest that CLICs play important roles in cellular physiology and pathology including heart. However, the pathophysiological roles of CLICs in cardiac ventricular myocytes including human heart remain unclear.

We aimed to investigate transcriptional changes of single ventricular myocytes in ion channels, particularly CLICs, during pressure overload‐induced hypertrophy and heart failure. We analyzed gene expression in cardiomyocytes from a TAC mouse model and patients with dilated cardiomyopathy (DCM) using single‐cell RNA sequencing (RNA‐seq) to elucidate the molecular mechanisms driving cardiac remodeling (hypertrophy and heart failure) by CLICs.

## 2 MATERIALS AND METHODS

### 2.1 Animal models

For the TAC model, we used male C57BL/6J mice (8 weeks old) to investigate the role of ion channels, particularly CLICs, in pressure overload‐induced cardiac remodeling hypertrophy and in heart failure (Nomura et al., 2018). TAC induces pressure overload in the left ventricle, mimicking hypertension or aortic stenosis, resulting in left ventricular hypertrophy and subsequently heart failure. The mice were classified into the following groups based on the time points at which they were sacrificed after TAC surgery (Day 3 and Weeks 1, 2, 4, 8 post‐TAC) (Fujiu et al., 2017; Houser et al., 2012; Nomura et al., 2018).

- Day 3 and Weeks 1 and 2 post‐TAC: Hypertrophy phase
- Weeks 4 and 8 post‐TAC: Late‐stage pressure overload, corresponding to stages associated with heart failure in prior studies.

Single ventricular myocytes were isolated from the left ventricular free wall in each group. Data from TAC‐operated mice were compared to those from sham‐operated control mice to identify specific gene expression changes associated with pressure overload.

### 2.2 Ethics and compliance

All animal experiments were approved by the Ethics Committee for Animal Experiments of the University of Tokyo (Approval number: RAC150001) and were conducted in strict accordance with animal experiment guidelines. Male C57BL/6J mice were obtained from CLEA Japan and maintained in a specific pathogen‐free facility. All mice were housed under a 12‐h/12‐h light/dark cycle at 24°C ± 1°C and were provided with standard mouse food (CE‐2; 59% carbohydrate and 12% soybean‐based fat of total energy, 344 kcal/100 g; CLEA Japan), and water ad libitum. At designated endpoints, animals were deeply anesthetized with isoflurane until loss of pedal and corneal reflexes, followed by cervical dislocation as a secondary method, in accordance with institutional guidelines.

All human experiments were approved by the Ethics Committee of the University of Tokyo (Approval number: G‐10032, approved on July 21, 2015). All procedures were conducted in accordance with the Declaration of Helsinki. Written informed consent was obtained from all participants prior to their inclusion in the study, and the privacy rights of the study participants were observed throughout the study.

### 2.3 Isolation of single cardiomyocytes

Mouse cardiomyocytes were isolated using Langendorff perfusion from the left ventricular free wall after sham operation and at 3 days and 1, 2, 4, and 8 weeks after TAC. Subsequently, we manually picked up these cardiomyocytes into 96‐well plates containing Smart‐seq2 lysis buffer using a 0.2–2 μL micropipette (sample volume, 0.5 μ L; Picelli et al., 2014).

Furthermore, single ventricular myocytes were obtained from patients with DCM undergoing heart surgery and from healthy donor hearts. We isolated cardiomyocytes from fresh myocardial tissue using the enzymatic digestion protocol established by Coppini et al. (2014) and Picelli et al. (2014). In brief, tissue samples were subjected to collagenase‐based perfusion (collagenase Type II, Worthington, Lakewood, NJ) and gentle mechanical dissociation to enrich the viable rod‐shaped myocytes.

Heart samples were collected from healthy participants (*n* = 2) and patients with DCM (*n* = 10). Normal heart tissues were obtained immediately after death due to non‐cardiac causes. DCM heart tissues were obtained during left ventricular assist device implantation.

### 2.4 Single‐cell RNA‐seq

After manual single‐cell pickup, we performed reverse transcription and PCR following the Smart‐seq2 protocol, generating single‐cell cDNA libraries (Nomura et al., 2018). Reverse transcription was performed using *SuperScript II Reverse Transcriptase* (Thermo Fisher Scientific, Cat# 18064014) with Anchored oligo‐dT primer and Template Switching Oligo. PCR was performed with *KAPA HiFi HotStart ReadyMix* (Roche/KAPA Biosystems, Cat# KK2602) and ISPCR primer. The efficiency of reverse transcription was assessed by examining the cycle threshold values of control genes (*Tnnt2*, *Cox6a2*) from quantitative real‐time polymerase chain reaction (qRT‐PCR) using a CFX96 Real‐Time PCR Detection System (Bio‐Rad) and by examining the distribution of the lengths of cDNA fragments using a LabChip GX (Perkin Elmer) and/or TapeStation 2200 (Agilent Technologies). The following primer sets were used for qRT‐PCR: Tnnt2 mRNA forward, TCCTGGCAGA GAGGAGGAAG; Tnnt2 mRNA reverse, TGCAGGTCGA ACTTCTCAGC; Cox6a2 mRNA forward, CGTAGCCCTC TGCTCCCTTA; Cox6a2 mRNA reverse, GGATGCGGAG GTGGTGATAC. A cycle threshold value of 25 was set as the threshold. Libraries that passed quality control were subjected to paired‐end 51‐bp RNA‐seq on a HiSeq 2500 in rapid mode. Following the Smart‐seq2 protocol, sequencing was performed to achieve a depth of 4 million reads per single cell. Reads were mapped to the mouse genome (mm9) using Bowtie (version 1.1.1) with the parameters “‐S ‐m 1 ‐l 36 ‐n 2 mm9” (Langmead et al., 2009). RPKM was calculated with reads mapped to the nuclear genome using DEGseq (version 1.8.0) (Wang et al., 2010).

### 2.5 RNA‐seq data analysis

The RNA‐seq data were processed using bioinformatics pipelines. Differential expression analysis was conducted to compare gene expression levels between the TAC and sham groups, and between patients with DCM and healthy donors. Genes with a fold change ≥2 and an adjusted *p*‐value <0.05 were considered significantly differentially expressed. Furthermore, we performed Kyoto Encyclopedia of Genes and Genome (KEGG) pathway analysis using the Database for Annotation, Visualization and Integrated Discovery (DAVID) bioinformatics tool (https://davidbioinformatics.nih.gov/) to assess the broader biological pathways involved in cardiac remodeling. This analysis focused on identifying the key pathways associated with CLIC expression, particularly those related to ECM focal adhesion pathways and actin cytoskeleton regulation. These pathways were investigated to understand the impact of CLICs on both the structural and electrical remodeling of the heart under chronic pressure overload.

### 2.6 Correlation network construction

Pearson correlation coefficients (*r*) were calculated for gene expression levels across samples to construct the correlation networks. Genes were categorized as either significantly upregulated or downregulated based on their fold change and statistical significance (*p* < 0.05).

The following thresholds were applied for the TAC model:

- CLIC1: *r* > 0.30
- CLIC4: *r* > 0.30
- CLIC5: *r* > 0.25

The thresholds for DCM were as follows:

- CLIC1: *r* > 0.30
- CLIC4: *r* > 0.25
- CLIC5: *r* > 0.20

These thresholds were selected to include biologically relevant interactions while minimizing noise. Separate networks were constructed for the upregulated and downregulated genes, highlighting distinct interaction patterns. The resulting networks were visualized using Cytoscape (version 3.10.3; https://cytoscape.org). Nodes represent individual genes, whereas edges indicate significant correlations. Node size and color were adjusted to emphasize attributes, such as expression changes or pathway involvement.

### 2.7 Statistical analysis

All statistical analyses were performed using GraphPad Prism (GraphPad Software, La Jolla, CA, USA) and R software. Continuous data are expressed as mean ± standard deviation. Statistical comparisons between the six groups (TAC model) were performed using the Kruskal–Wallis test. After a significant result from the test, post hoc tests were performed using a pairwise Mann–Whitney *U* test with Bonferroni correction, and *p* < 0.05 was considered significant. Comparison between two groups (control vs. DCM) was performed using the Mann–Whitney *U* test, and *p* < 0.05 was considered significant.

## 3 RESULTS

### 3.1 Fetal gene reactivation in the TAC model

**FIGURE 1:**
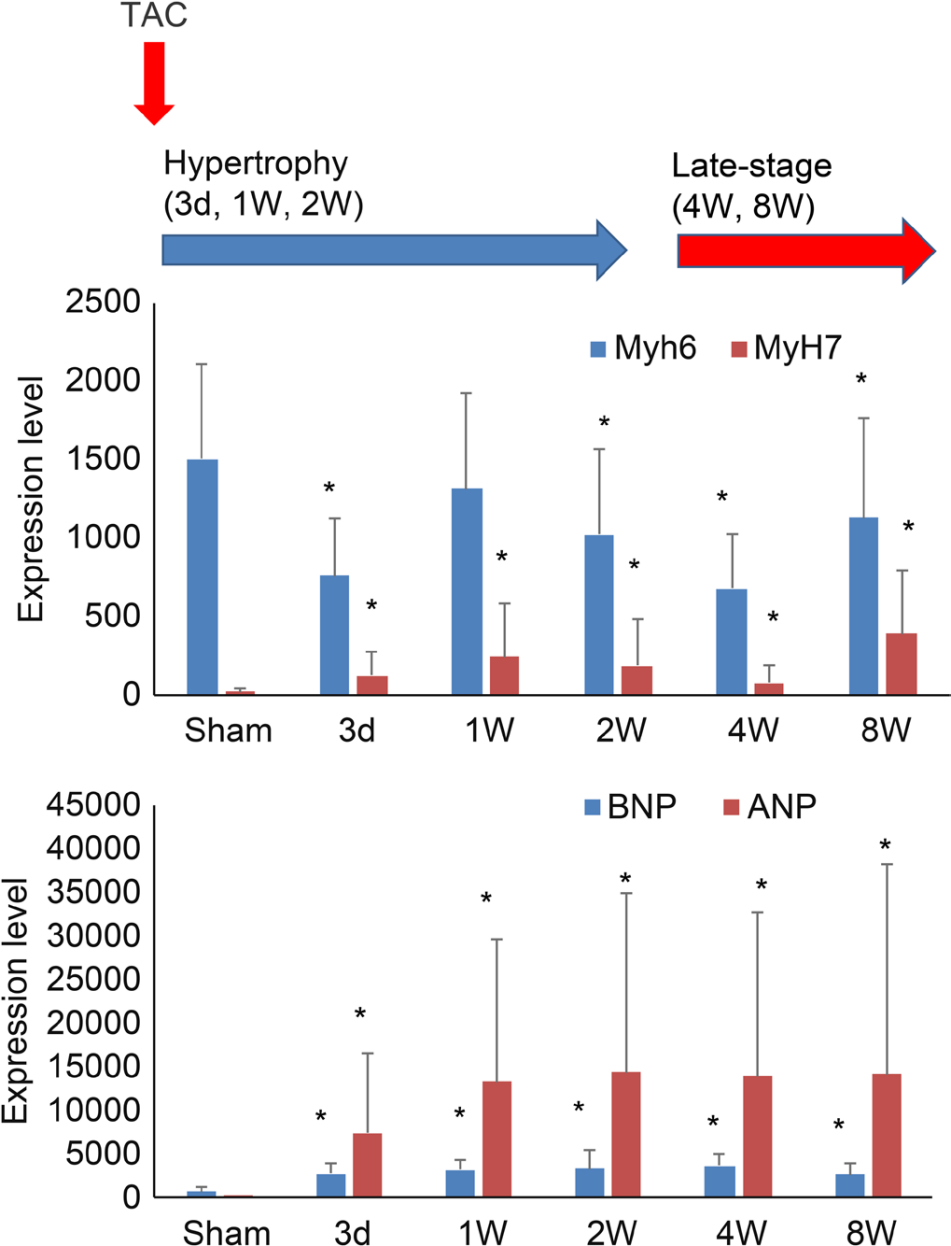
Fetal gene reactivation in the TAC model. Sham (*n* = 64 cells). Hypertrophy phase; Day 3 (*n* = 58 cells), 1 week (*n* = 82 cells), and 2 week (*n* = 61 cells). Late‐stage post TAC; 4 week (*n* = 73 cells) and 8 week (*n* = 58 cells). Effects of TAC on Myh6, Myh7, atrial natriuretic peptide, and brain natriuretic peptide mRNA levels in the TAC model. *p < 0.05 versus Sham. TAC, transverse aortic constriction.

The TAC model revealed distinct temporal changes in fetal gene markers in ventricular myocytes during hypertrophy and heart failure progression. *Myh7* (myosin heavy chain β), atrial natriuretic peptide, and brain natriuretic peptide were significantly upregulated from Day 3 post‐TAC, persisting through Weeks 1, 2, 4, and 8. In contrast, *Myh6* (myosin heavy chain *α*) was significantly downregulated during these time points, highlighting a switch from adult to fetal gene expression profiles. These findings suggest that early and sustained activation of the fetal gene program occurs in response to pressure overload. This reactivation reflects a pathological shift toward a fetal‐like transcriptional state, which is indicative of maladaptive remodeling and impaired cardiac function under chronic mechanical stress (Figure 1).

### 3.2 Significant remodeling of ion channels in the TAC model

Single‐cell RNA‐seq enables the detection of transcriptional changes in individual cardiomyocytes and provides a high‐resolution view of gene expression dynamics in response to pressure overload. Among the 23,355 genes, 212 related to ion channels and transporters were analyzed (Table 1). These were:

- Ion channels, including potassium (K^+^), sodium (Na^+^), calcium (Ca^2+^), and chloride (Cl^−^)
- CLICs, particularly *CLIC1*, *CLIC4*, and *CLIC5*—hypothesized to play critical roles in cardiac remodeling via their involvement in ion transport and mitochondrial function, as previously reported (Bayeva et al., 2013; Gururaja Rao et al., 2020; Ponnalagu et al., 2016)
- Transporters and exchangers, such as the Na^+^/Ca^2+^ exchanger (*NCX1*), which are key for maintaining calcium homeostasis in cardiomyocytes

**TABLE 1:** Analyzed ion channel and transporter‐related genes.

TRP and related mRNA	Trpv1 Trpv2 Trpv3 Trpv4 Trpv5 Trpv6 Trpa1 Trpc1Trpc2 Trpc3 Trpc4 Trpc4ap Trpc5 Trpc6 Trpc7 Trpm1 Trpm2 Trpm3 Trpm4 Trpm5 Trpm6 Trpm7 Trpm8 Trpml1 Trpml2 Trpml3 Pkd1 Pkd1I2 Pkd1I3 Pkd2 Pkd2I1 PkdI2 Orai1 Orai2
K^+^ channel and related mRNA	Kcna1 Kcna10 Kcna2 Kcna3 Kcna4 Kcna5 Kcna6 Kcna7 Kcnab1 Kcnab2 Kcnab3 Kcnb1 Kcnb2 Kcnc1 Kcnc2 Kcnc3 Kcnc4 Kcnd1 Kcnd2 Kcnd3 Kcne1 Kcne1l Kcne2 Kcne3 Kcne4 Kcnf1 Kcng1 Kcng2 Kcng3 Kcng4 Kcnh1 Kcnh2 Kcnh3 Kcnh4 Kcnh5 Kcnh6 Kcnh7 Kcnh8 Kcnip1 Kcnip2 Kcnip3 Kcnip4 Kcnj1 Kcnj10 Kcnj11 Kcnj12 Kcnj13 Kcnj14 Kcnj15 Kcnj16 Kcnj2 Kcnj3 Kcnj4 Kcnj5 Kcnj6 Kcnj8 Kcnj9 Kcnk1 Kcnk10 Kcnk12 Kcnk13 Kcnk15 Kcnk16 Kcnk18 Kcnk2 Kcnk3 Kcnk4 Kcnk5 Kcnk6 Kcnk7 Kcnk9 Kcnma1 Kcnmb1 Kcnmb2 Kcnmb3 Kcnmb4 Kcnn1 Kcnn2 Kcnn3 Kcnn1 Kcnq1 Kcnq1ot1 Kcnq2 Kcnq3 Kcnq4 Kcnq5 Kcnrg Kcns1 Kcns2 Kcns3 Kcnt1 Kcnt2 Kcnu1 Kcnv1 Kcnv2 Abcc9
Ca^2+^ channel and related mRNA	Cacna1b Cacna1c Cacna1d Cacna1g Cacna1h Cacna1s Cacna2d Cacna2d2 Cacna2d3 Cacnb1 Cacnb2
Na^+^ channel	Scn5a
Na^+^/Ca^2+^ exchanger	Slc8a1
connexin‐43	Gja1
Na^+^/H^+^ exchanger	Slc9a1
Hyperpolarization‐activated cyclic nucleotide–gated (HCN) channels	Hcn1 Hcn2 Hcn3 Hcn4
Na^+^, K^+^‐ATPase subunit mRNA	Atp1a1 Atp1a2 Atp1a3 Atp1a4 Atp1b1 Atp1b2 Atp1b3 Atp1b4
Ca^2+^‐ATPase mRNA	Atp2a1 Atp2a2 Atp2a3 Atp2b1 Atp2b2 Atp2b3 Atp2b4 Atp2c1 Atp2c2
Anion channel mRNA	Clcn1 Clcn2 Clcn3 Clcn4‐2 Clcn5 Clcn6 Clcn7 CFTR Ano1 Ano2 Ano3 Ano4 Ano5 Ano6 Ano7 Ano8 Ano9 Ano10 Clca1 Clca2 Clca3 Clca4 Clca5 Clca6 Clcc1 Best1 Best2 Vdac1 Vdac2 Vdac3 Clic1 Clic2 Clic3 Clic4 Clic5 Clic6
Aquaporin mRNA	Aqp1 Aqp2 Aqp3 Aqp4 Aqp5 Aqp6 Aqp7 Aqp8 Aqp9 Aqp11 Aqp12

**FIGURE 2:**
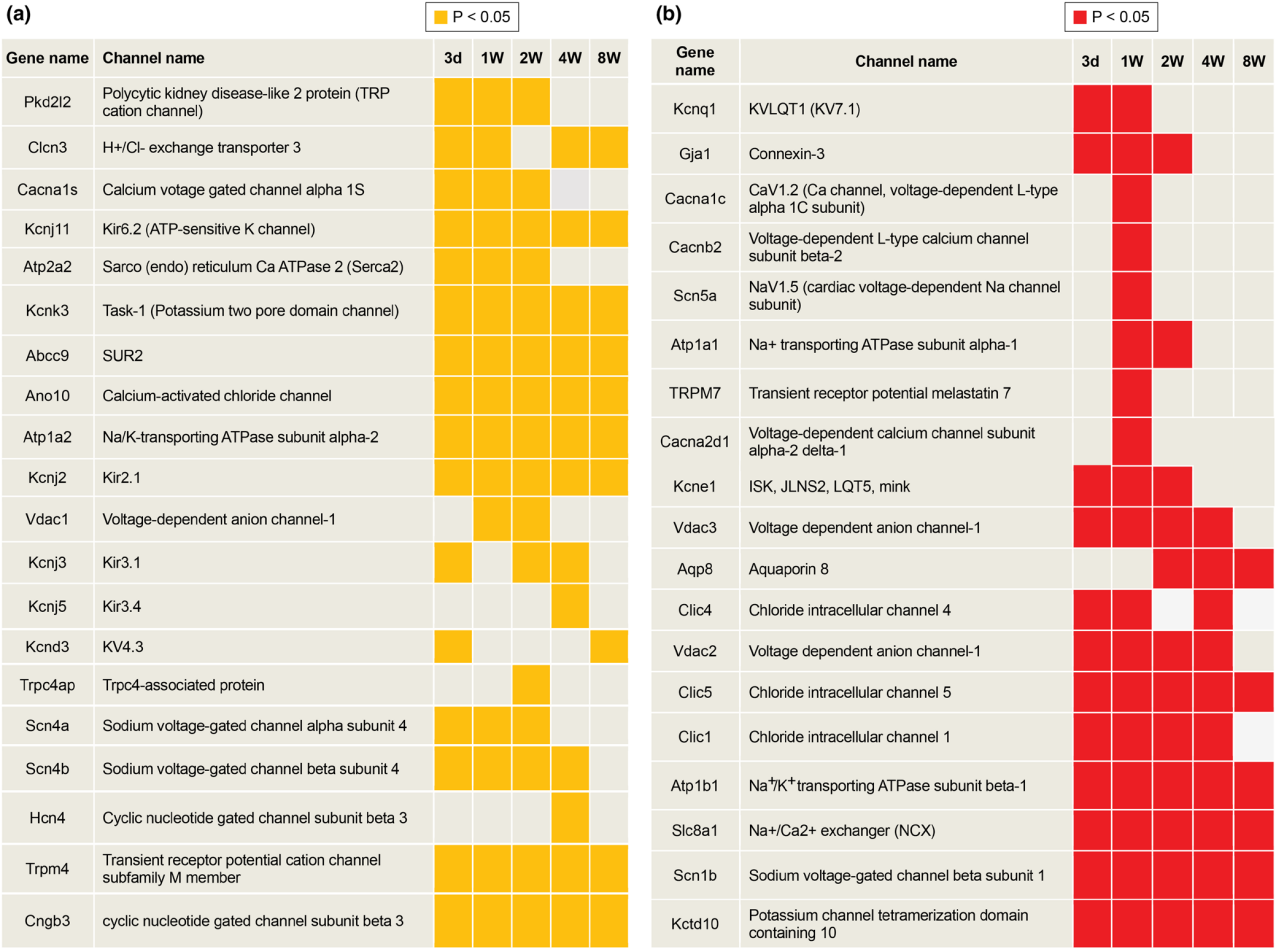
Temporal changes in gene expression levels across the hypertrophic and heart failure phases in the TAC model. Yellow (Section a): Represents significantly decreased expression (*p* < 0.05). Red (Section b): Represents significantly increased expression. *p* < 0.05. TAC, transverse aortic constriction. Sham (*n* = 64 cells). Hypertrophy phase; Day 3 (*n* = 58 cells), 1 week (*n* = 82 cells), and 2 week (*n* = 61 cells). Heart failure phase; 4 week (*n* = 73 cells) and 8 week (*n* = 58 cells).

Significant remodeling of ion channels was observed in the TAC model (Figure 2). A detailed analysis of ion channel and transporter‐related genes revealed substantial upregulation and downregulation of several genes.

### 3.3 Downregulated genes

Significant downregulation was observed for specific ion channels and transporters (Figure 2a):

- Task‐1 (*Kcnk3*): A two‐pore domain K^+^ channel involved in setting the resting membrane potential
- Kir6.2 (*Kcnj11*): An ATP‐sensitive inward rectifier K^+^ channel, crucial for membrane potential regulation under metabolic stress
- Kir2.1 (*Kcnj2*): Stabilizes the resting membrane potential and contributes to action potential stability
- Kv4.3 (*Kcnd3*): A transient outward K^+^ channel regulating the repolarization phase of the action potential.

The increased expression of these genes suggests enhanced repolarization capacity and altered excitability, which are characteristics of hypertrophic and failing hearts.

### 3.4 Upregulated genes (Figure 2, Panel b)

Several key ion channels and transporters were significantly upregulated during progression (Figure 2b):

- Slc8a1 (*Ncx1*): A sodium‐calcium exchanger critical for calcium extrusion that may contribute to impaired calcium handling and contractility.
- *CLIC4* and *CLIC5*: Chloride intracellular channels implicated in ion transport and mitochondrial function, both of which exhibited significant increases.

Figure 2 shows the dynamic remodeling of ion channels and transporter‐related genes, providing insights into the molecular adaptations to pressure overload in the TAC model, which reflects changes in cardiac excitability, calcium handling, and mitochondrial health during disease progression.

### 3.5 Correlation networks of upregulated and downregulated genes in the TAC model

**FIGURE 3:**
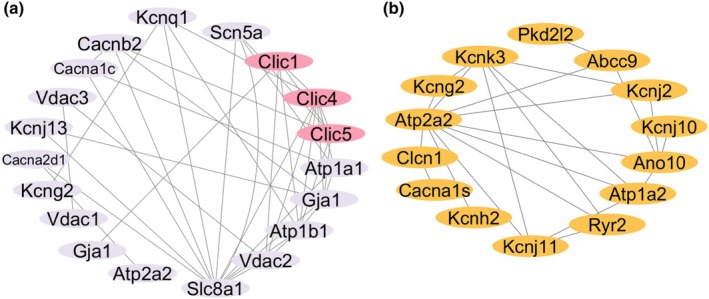
Correlation networks of upregulated and downregulated genes in the TAC model. (a) Correlation among increased genes in the TAC model. This figure represents the correlation network of genes with increased expression levels, particularly highlighting those associated with CLIC1, CLIC4, and CLIC5. Lines connect genes that have a statistically significant relationship, demonstrating potential functional interactions or co‐regulation. (b) Correlation among decreased genes in the TAC model. This figure represents the correlation network among genes with decreased expression levels. Lines connect genes with significant associations, illustrating coordinated downregulation pathways or related physiological processes. CLIC, chloride intracellular channel; TAC, transverse aortic constriction.

Pearson's correlation analysis was performed to investigate gene expression patterns in the TAC model, revealing distinct interaction networks among the upregulated and downregulated genes (Figure 3).

#### 3.5.1 Upregulated gene correlation network

Genes with increased expression in the TAC model demonstrated significant correlations, particularly with *CLIC1*, *CLIC4*, and *CLIC5*. The upregulation of these genes, including those involved in ion transport, cytoskeletal remodeling, and cellular signaling, suggests a coordinated transcriptional response to chronic cardiac stress. The correlation network highlighted potential functional interactions and co‐regulation among these genes, contributing to structural and functional alterations in cardiomyocytes and cardiac remodeling, with upregulated *CLIC*s shown alongside other upregulated genes (Figure 3a).

#### 3.5.2 Downregulated gene correlation network

Conversely, genes with decreased expression in the TAC model formed distinct correlation networks. These genes were primarily associated with ion transport and metabolic regulation and exhibited significant interactions, indicating coordinated downregulation of pathways. The network highlighted gene interactions contributing to altered metabolic and ion transport functions in cardiomyocytes, suggesting impaired cellular resilience and contractility (Figure 3b).

### 3.6 CLICs and mitochondrial dysfunction

**FIGURE 4:**
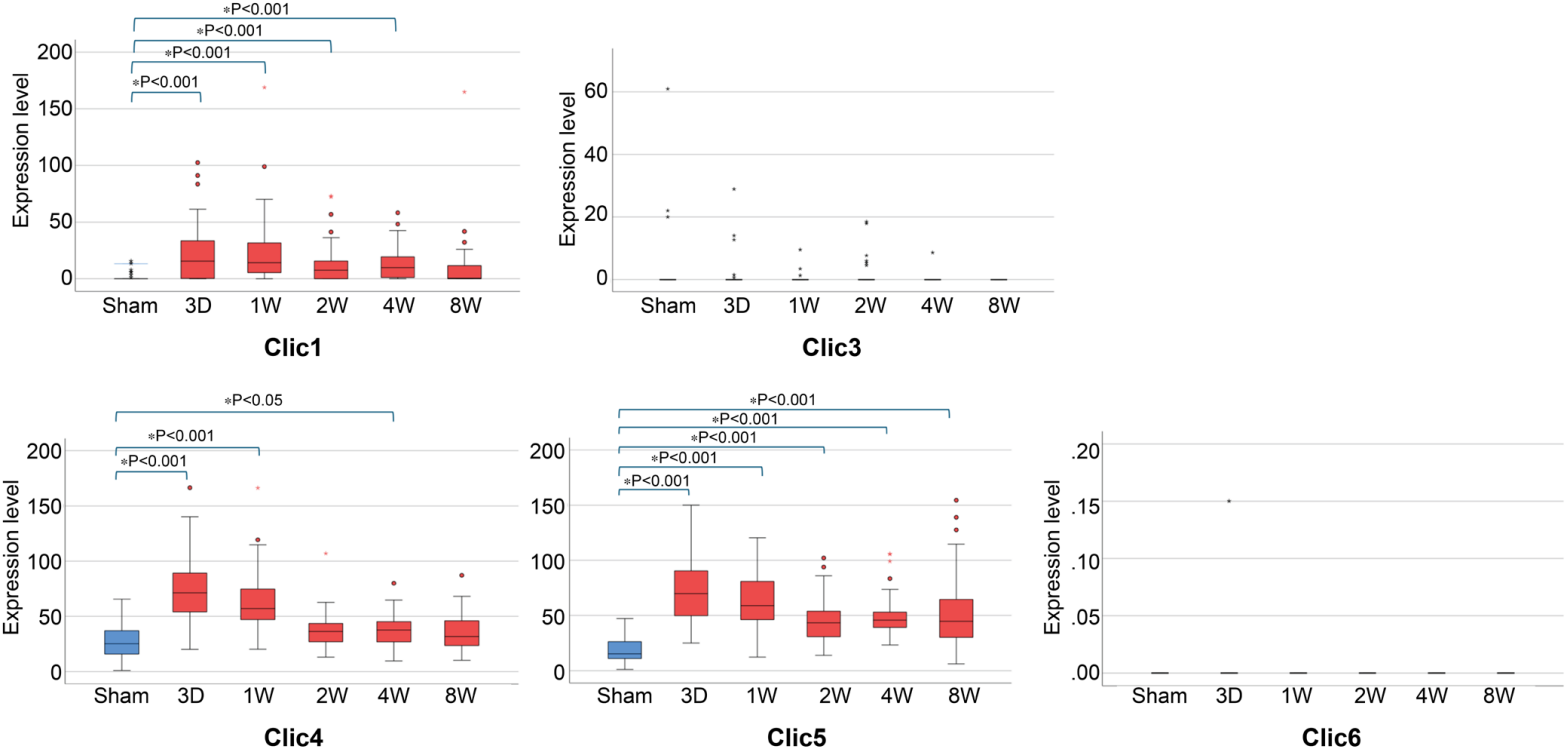
Changes of mRNA expression level of CLIC family genes in the TAC model. This figure displays the expression levels of CLIC family genes (CLIC1 and CLIC3‐6) in the TAC model, analyzed across different time points: sham (*n* = 64 cells), 3 days (*n* = 58 cells), 1 week (*n* = 82 cells), 2 weeks (*n* = 61 cells), 4 weeks (*n* = 73 cells), and 8 weeks (*n* = 58 cells) post‐TAC. The data reveal distinct temporal patterns of gene expression associated with the progression from hypertrophy to heart failure. Statistical Analysis: Differences in gene expression between groups were assessed using the Kruskal–Wallis test (*p < 0.05). Significant comparisons are indicated by asterisks. CLIC, chloride intracellular channel; TAC, transverse aortic constriction.

Among *CLIC*s, *CLIC1*, *CLIC4*, and *CLIC5* were significantly upregulated in the TAC model (Figure 4). *CLIC1* mRNA expression levels significantly increased in the early hypertrophy phase and remained elevated at Week 4. *CLIC3* mRNA expression remained low and showed no significant changes over time, indicating its limited involvement in the TAC model. A marked increase in *CLIC4* mRNA was observed from Day 3 post‐TAC to Week 4, followed by stabilization at Week 8, suggesting its role in early‐to mid‐phase remodeling. Notably, the expression of *CLIC5* mRNA significantly increased on Day 3 post‐TAC and remained elevated throughout all phases. No significant changes in *CLIC6* mRNA were detected across all time points, suggesting its limited involvement in the TAC model. These results suggest that genes in the CLIC family may play differential roles in cardiac remodeling, reflecting their distinct contributions to the pathological response in the TAC model.

### 3.7 Pathway analysis of CLICs and related genes

**FIGURE 5:**
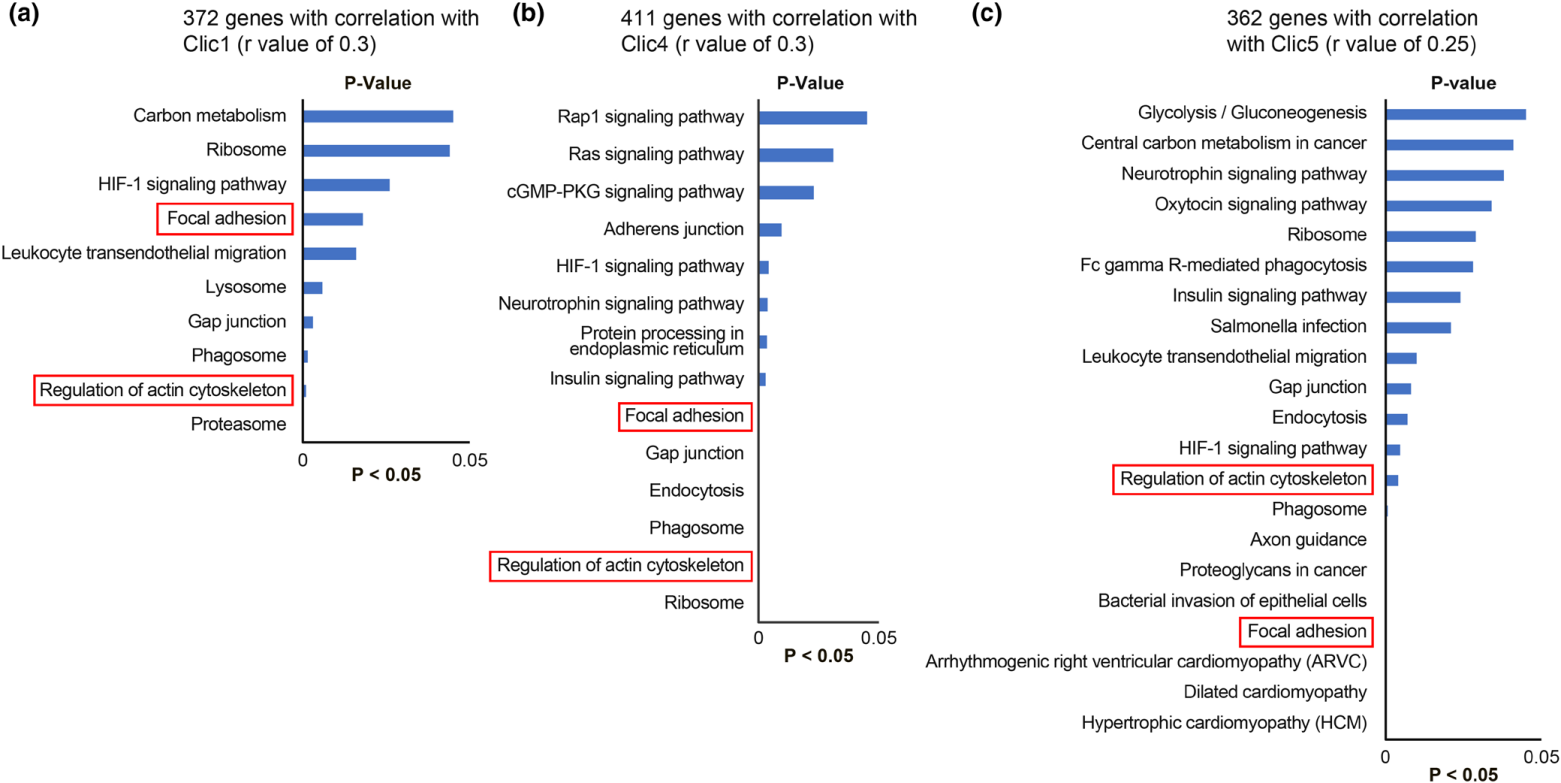
KEGG pathway analysis of genes correlated with CLIC1, CLIC4, and CLIC5 in the TAC model. (a) CLIC1; (b) CLIC4; (c) CLIC5. KEGG pathway analysis identified significant enrichment of genes correlated with CLIC1, CLIC4, and CLIC5 in pathways related to focal adhesion, actin cytoskeleton regulation, and ECM remodeling. These results highlight the potential role of CLIC proteins in cardiac fibrosis and cytoskeletal reorganization under pressure overload conditions. Pathways with *p* < 0.05 are displayed. CLIC, chloride intracellular channel; ECM, extracellular matrix; KEGG, Kyoto Encyclopedia of Genes and Genome; TAC, transverse aortic constriction.

KEGG pathway analysis using the DAVID bioinformatics tool identified a significant enrichment of genes correlated with *CLIC1*, *CLIC4*, and *CLIC5* in pathways related to focal adhesion, ECM remodeling, and actin cytoskeleton regulation. These findings suggest that CLIC proteins play a crucial role in structural remodeling under pressure overload (Figure 5). Pathways associated with focal adhesion and actin cytoskeleton regulation were significantly enriched in *CLIC1*‐correlated genes (*r* > 0.3; 372 genes), indicating that CLIC1 may be involved in ECM remodeling and cytoskeletal reorganization. Similarly, 411 *CLIC4*‐correlated genes (*r* > 0.3) were significantly associated with focal adhesion, actin cytoskeleton regulation, and neurotrophin signaling pathways, suggesting that CLIC4 may contribute to both ECM remodeling and cytoskeletal stability in response to mechanical stress. Enriched pathways for *CLIC5*‐correlated genes (*r* > 0.25, 362 genes) included focal adhesion, actin cytoskeleton regulation, and immune‐related pathways, such as HIF‐1 signaling and Fc gamma receptor‐mediated phagocytosis. These findings indicate that CLIC5 plays a role in mechano‐transduction and inflammatory responses during cardiac remodeling. Overall, these results support the hypothesis that CLIC proteins are key regulators of cardiac structural integrity and cytoskeletal remodeling, linking mechanical stress to molecular signaling events that drive heart failure progression.

### 3.8 Roles of CLIC1 in focal adhesion signaling pathways

**FIGURE 6:**
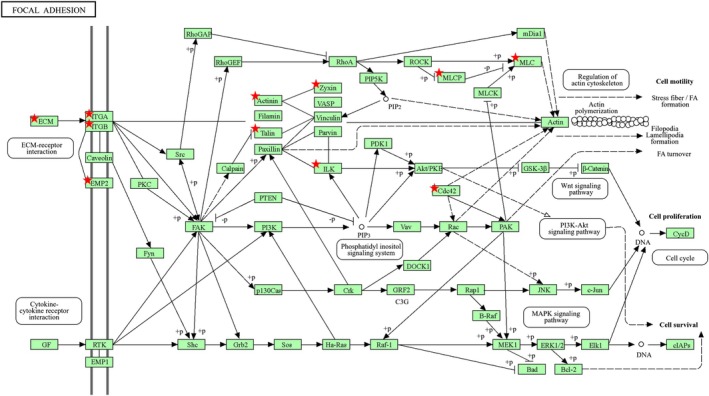
Role of CLIC1 in focal adhesion signaling pathways. The red asterisks (*) mark specific components that show a significant correlation with CLIC1 expression, emphasizing its potential role in modulating these pathways. CLIC, chloride intracellular channel.

Analysis of focal adhesion signaling pathways revealed that *CLIC1* was closely associated with key components involved in ECM interactions, cytoskeletal regulation, and downstream signaling cascades (Figure 6). Genes correlating with *CLIC1* expression were significantly enriched in focal adhesion‐related pathways, suggesting a role in maintaining cellular adhesion, motility, and structural stability under mechanical stress. *CLIC1* showed a strong correlation with integrin genes (*ITGA*/*ITGB*) and ECM components, indicating its involvement in ECM‐receptor interactions and focal adhesion stabilization. Additionally, *CLIC1* was associated with genes for cytoskeletal regulatory proteins such as paxillin, vinculin, and talin, which are essential for actin filament organization and stress fiber formation. These interactions suggest that *CLIC1* contributes to cytoskeletal remodeling, enabling cellular adaptation to mechanical stress. Furthermore, the PI3K‐Akt and MAPK signaling pathways, which play crucial roles in cell survival, proliferation, and migration, were significantly linked with *CLIC1* expression. Moreover, components such as ILK and CDC42, which regulate cell shape and motility, exhibited strong correlations with *CLIC1*. These findings suggest that CLIC1 facilitates cellular adaptation by modulating cytoskeletal dynamics and focal adhesion turnover, thereby influencing cardiac remodeling under pathological conditions.

**FIGURE 7:**
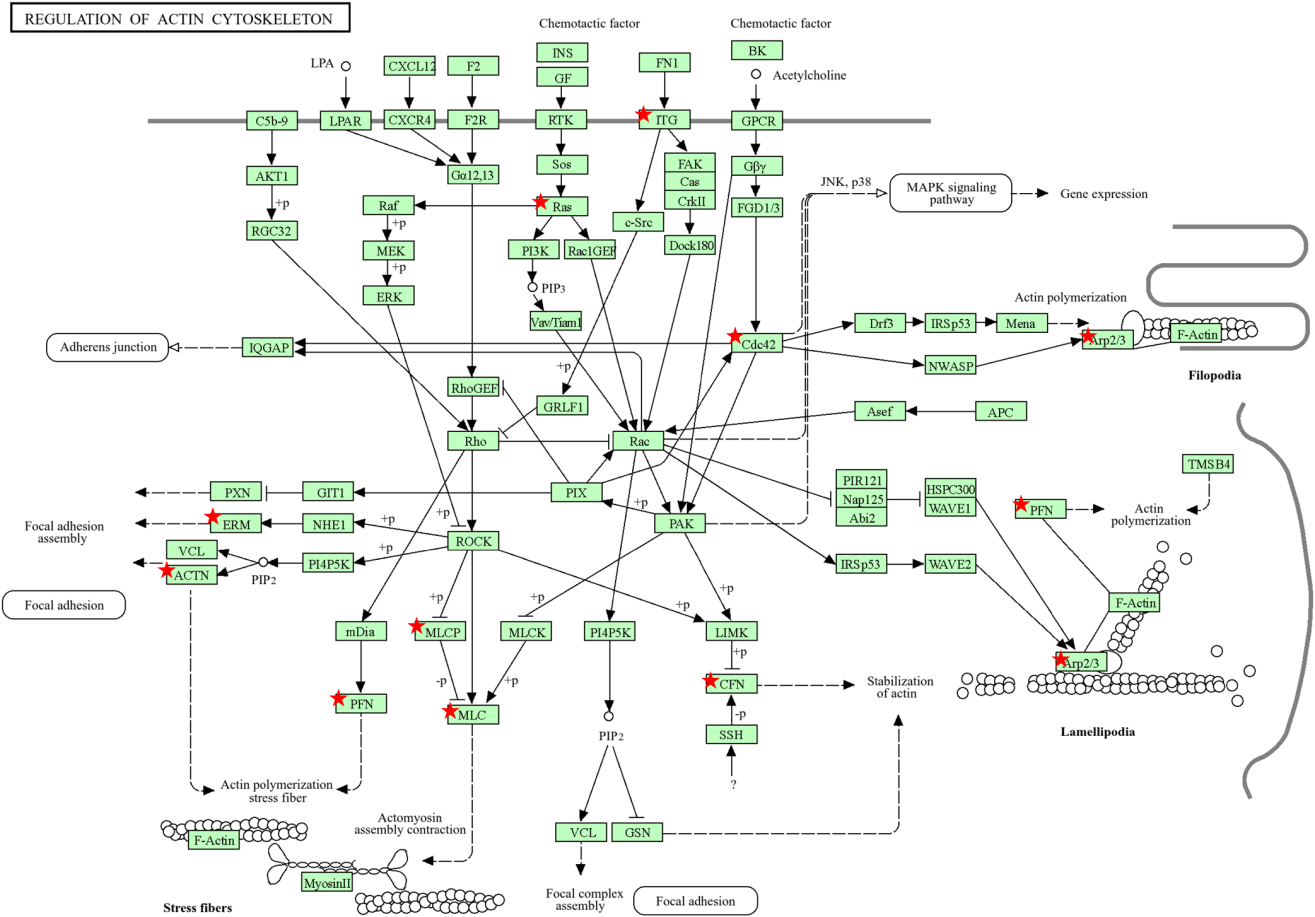
Role of CLIC1 in the regulation of actin cytoskeletal pathways. The red asterisks (*) mark specific components that show a significant correlation with CLIC1 expression, emphasizing its potential role in modulating these pathways. CLIC, chloride intracellular channel.

Furthermore, *CLIC1* was closely associated with key regulators of the actin cytoskeleton, influencing processes such as actin polymerization, filament branching, and cytoskeletal stabilization (Figure 7). Pathway analysis revealed a significant correlation between *CLIC1* expression and genes involved in actin cytoskeletal organization, suggesting its role in cellular structural remodeling under mechanical stress conditions.

The genes of key actin‐regulating proteins, including cofilin, profilin, and the Arp2/3 complex, were significantly associated with *CLIC1* expression (Figure 7). These proteins are essential for actin filament remodeling and branching, which maintain cell shape and motility. The correlation between CLIC1 and these regulatory elements suggests that CLIC1 may facilitate cytoskeletal reorganization in response to external stimuli. Additionally, focal adhesion proteins such as paxillin (PXN) and vinculin (VCL) exhibited strong correlations with CLIC1, indicating their involvement in linking the actin cytoskeleton to the cell membrane. This interaction is crucial for stress fiber formation and cellular adhesion, particularly under pathological conditions that require structural adaptation. Furthermore, upstream signaling regulators, including Rho GTPases (e.g., CDC42 and Rac) and kinases, such as ROCK and LIMK, were additionally identified as CLIC1‐associated components. These signaling molecules play a fundamental role in actin polymerization and cytoskeletal stabilization, further supporting the hypothesis that CLIC1 influences actin cytoskeletal dynamics by interacting with these pathways. These findings suggest that CLIC1 plays a crucial role in actin cytoskeletal remodeling, contributing to cellular motility, adhesion, and mechanical stability. Its involvement in actin cytoskeletal regulation may be particularly relevant to cardiac remodeling processes, in which cytoskeletal reorganization is a key adaptive response to mechanical stress.

### 3.9 Gene interaction networks of CLIC1, CLIC4, and CLIC5 in the TAC model

Gene correlation networks were constructed using the TAC model to investigate the functional roles of CLIC1, CLIC4, and CLIC5 in myocardial remodeling, revealing distinct interaction patterns that link CLIC proteins to focal adhesions, actin cytoskeleton regulation, and ECM remodeling.

**FIGURE 8:**
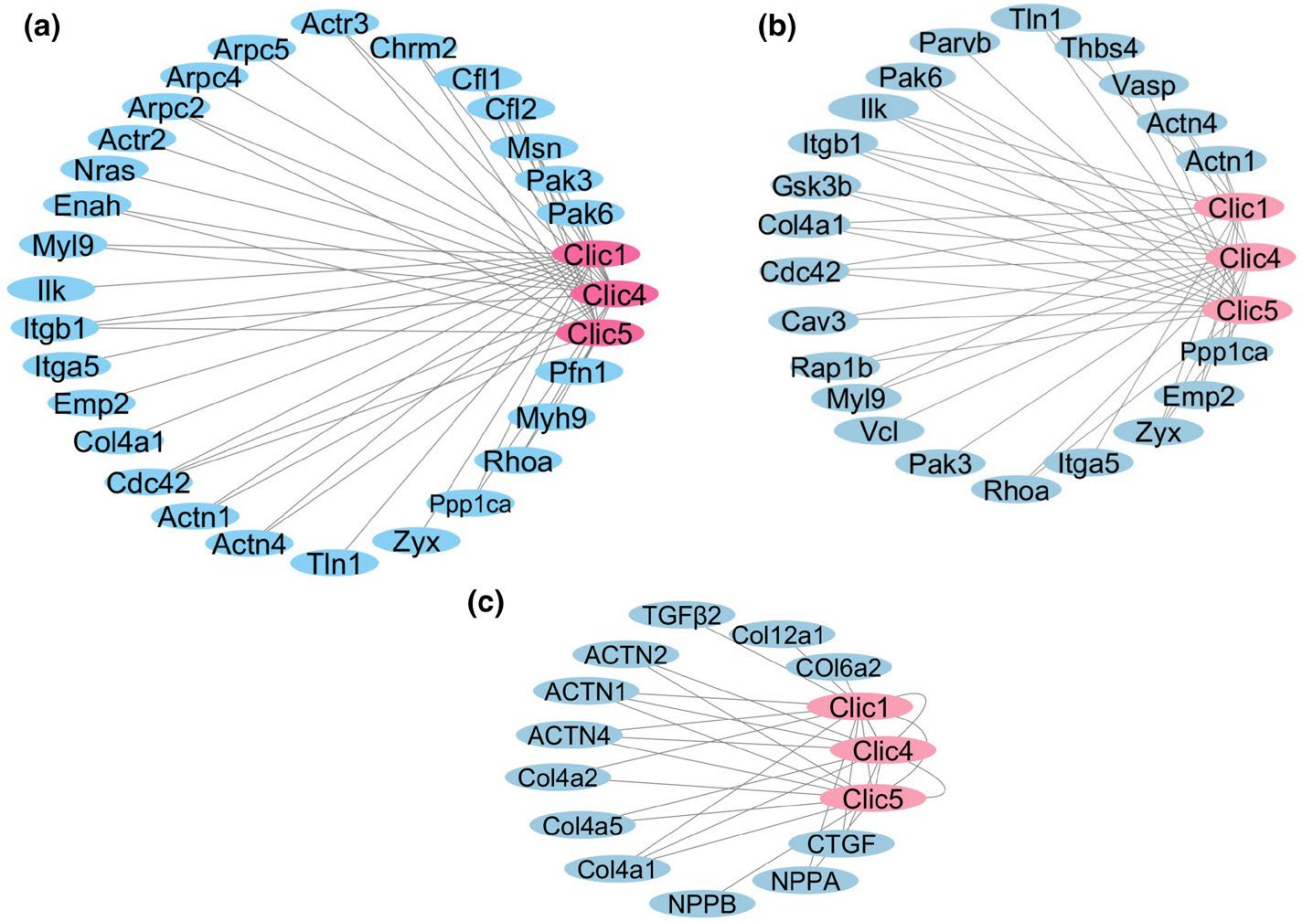
Correlation networks of CLIC1, CLIC4, and CLIC5 with focal adhesion, actin cytoskeleton, and fibrotic remodeling in the TAC model. (a) Gene interaction network of CLIC1, CLIC4, and CLIC5 in the focal adhesion pathway. CLICs are central nodes associated with key adhesion‐related genes, indicating their involvement in ECM‐cell interactions and cytoskeletal stability. (b) Network of genes correlated with CLIC1, CLIC4, and CLIC5 in actin cytoskeleton regulation. These interactions highlight the role of CLICs in actin remodeling and cytoskeletal organization. (c) Interaction between collagens, actinins, and CLICs in the TAC model. CLICs are associated with ECM components, actinins, and fibrotic regulators, emphasizing their role in fibrosis and structural remodeling under mechanical stress. CLIC, chloride intracellular channel; ECM, extracellular matrix; TAC, transverse aortic constriction.

#### 3.9.1 Focal adhesion pathway‐associated gene network (Figure 8a)

Network analysis demonstrated that *CLIC1*, *CLIC4*, and *CLIC5* function as central nodes, interacting with multiple genes involved in focal adhesion pathways. Genes such as *ITGA5*, *ITGB1*, *TLN1* (talin), *VCL* (vinculin), and *ZYX* (zyxin), whose products regulate focal adhesion complex formation, were strongly correlated with CLIC proteins. These findings suggest that CLICs play a role in ECM‐cell interactions, cytoskeletal organization, and cellular adhesion, particularly under conditions of mechanical stress.

#### 3.9.2 Actin cytoskeleton‐related gene network (Figure 8b)

The second correlation network focused on genes involved in actin cytoskeleton regulation. *CLIC1*, *CLIC4*, and *CLIC5* were significantly correlated with actinins (*ACTN1* and *ACTN4*), *CDC42*, and *ARPC2*, which are critical regulators of actin filament organization and branching. Additionally, genes involved in stress fiber formation and focal adhesion, such as *VCL*, *ZYX*, and *TLN1*, were included in the network, reinforcing the hypothesis that circRNAs contribute to cytoskeletal remodeling and cellular mechanical stability.

#### 3.9.3 CLIC interaction with collagens and fibrotic regulators (Figure 8c)

The third network examined correlations between classic collagen, actinins, and fibrotic regulators. *CLIC1*, *CLIC4*, and *CLIC5* were strongly associated with *COL4A1*, *COL4A2*, *COL4A5*, *COL6A2*, and *COL12A1*, which are the key components of ECM remodeling. These findings suggest that CLICs regulate myocardial ECM by modulating collagen expression and deposition.

Furthermore, *CLIC1* and *CLIC4* significantly correlated with *ACTN2* and *ACTN4*, which play crucial roles in linking the cytoskeleton to the sarcomere, supporting their involvement in maintaining cardiomyocyte structural integrity. In addition, CLIC proteins correlated with *CTGF*, *TGFβ2*, *NPPA*, and *NPPB*, whose products are known regulators of ECM remodeling and cardiac stress responses, suggesting that CLICs contribute to both ECM integrity and fibrotic progression in failing hearts.

### 3.10 Comparison between patients with DCM and healthy patients

#### 3.10.1 CLIC upregulation in DCM

**FIGURE 9:**
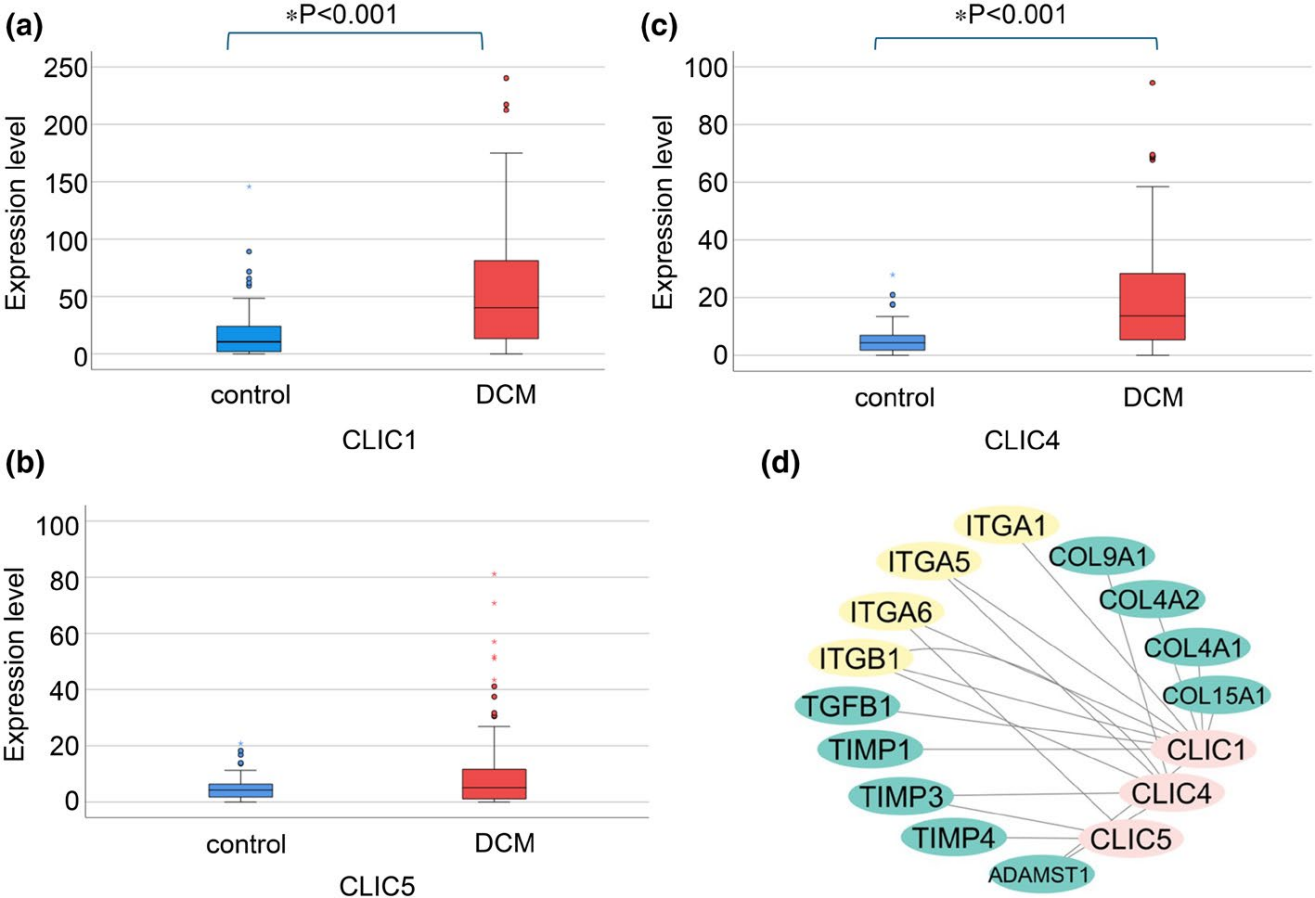
CLIC expression and ECM‐focal adhesion pathways in patients with DCM. (a) CLIC1 expression in DCM versus control. Box plot showing significant upregulation of CLIC1 in ventricular myocytes from patients with DCM (*n* = 217 cells) compared to healthy controls (*n* = 70 cells) (*p* < 0.05). (b) CLIC4 expression in DCM (*n* = 217 cells) versus control (*n* = 70 cells). Box plot illustrating significant upregulation of CLIC4 in DCM (*p < 0.05). (c) CLIC5 expression in DCM (*n* = 217 cells) versus control (*n* = 70 cells). Box plot showing a trend toward increased CLIC5 expression in DCM, but the change was not statistically significant. (d) Correlation network of CLICs with ECM and focal adhesion‐related genes in DCM. CLICs are central nodes interacting with genes involved in fibrosis, ECM remodeling, and cell adhesion, highlighting their role in structural remodeling and myocardial adaptation. CLIC, chloride intracellular channel; DCM, dilated cardiomyopathy; ECM, extracellular matrix.

Single‐cell RNA‐seq analysis of ventricular myocytes from patients with DCM revealed significant upregulation of *CLIC1* and *CLIC4* compared to healthy controls, suggesting that they play prominent roles in cardiac remodeling associated with DCM. In contrast, although *CLIC5* showed a trend toward increased expression, this change was not statistically significant. This observation indicated distinct regulatory patterns among CLIC family members, with *CLIC1* and *CLIC4* being the primary contributors to disease progression (Figure 9a–c).

#### 3.10.2 ECM‐focal adhesion and actin cytoskeleton pathways in DCM

Pathway analysis in patients with DCM identified significant enrichment of ECM‐focal adhesion and actin cytoskeleton regulation pathways, suggesting that CLICs contribute to pathological ECM remodeling. This remodeling process results in structural changes, ultimately contributing to the development of heart failure. Correlation analysis revealed that *CLIC1*, *CLIC4*, and *CLIC5* interacted with genes involved in ECM remodeling and focal adhesion, further supporting their roles in cardiac structural adaptation in DCM. Specifically, *ITGA5*, *ITGA6*, *ITGA1*, and *ITGB1*, which play key roles in cellular adhesion and myocardial integrity, were significantly associated with CLIC expression. In addition, collagen‐associated genes (*COL4A1*, *COL4A2*, *COL9A1*, and *COL15A1*) were strongly correlated with CLICs, suggesting that CLICs may regulate fibrosis and ECM remodeling in the failing myocardium. Furthermore, genes for fibrotic regulators, such as *TGFβ1*, *TIMP1*, *TIMP3*, *TIMP4*, and *ADAMTS1*, were linked to *CLIC* expression, reinforcing the hypothesis that CLICs contribute to fibrotic progression and ECM remodeling in human heart failure (Figure 9d). These findings show the possible role of CLICs in mediating ECM reorganization and alterations in cell adhesion, which are key features of fibrotic progression and pathological myocardial remodeling.

## 4 DISCUSSION

We show the possible role of CLICs in the progression of structural remodeling under pressure overload in cardiomyocytes in a mouse TAC model to induce cardiac hypertrophy and heart failure, and in patients with DCM. We used single‐cell RNA‐seq to demonstrate the significant upregulation of *CLIC1*, *CLIC4*, and *CLIC5* in a TAC mouse model and patients with DCM, underscoring their conserved roles in heart failure progression. In contrast, *CLIC5* expression showed an increasing trend in patients with DCM but did not reach statistical significance, suggesting a less prominent role than *CLIC1* and *CLIC4*. KEGG pathway analysis using DAVID for genes associated with *CLIC1*, *CLIC4*, and *CLIC5* revealed a strong association between the activation of focal adhesion and actin cytoskeleton regulation pathways in cardiomyocytes from a TAC mouse model and in patients with DCM.

Previous studies have reported the involvement of CLICs (*CLIC1*, *CLIC4*, and *CLIC5*) in AF, suggesting that these channels play a broader role in atrial remodeling beyond heart failure (Jiang et al., 2017). The present study is the first to report that CLICs may play essential roles in myocardial ECM remodeling, leading to myocardial hypertrophy and heart failure progression in the left ventricle under pressure overload and in DCM. Among CLICs, *CLIC1*, *CLIC4*, and *CLIC5* were significantly upregulated during the hypertrophic and heart failure phases of the TAC mouse model. *CLIC1* mRNA expression significantly increased in the early phase and remained elevated at Week 4 post‐TAC compared to that in sham mice. A marked increase in *CLIC4* mRNA expression was observed from Day 3 post‐TAC to Week 4, followed by stabilization at week 8, suggesting its role in early to mid‐phase remodeling. Notably, the expression of *CLIC5* mRNA significantly increased at day 3 post‐TAC and remained elevated throughout the hypertrophy and heart failure phases, indicating upregulation during disease progression. Single‐cell RNA‐seq analysis of single ventricular myocytes from patients with DCM also showed an increase in *CLIC1* and *CLIC4* mRNA expression compared to those from healthy donors. In contrast, *CLIC5* mRNA expression showed an increasing trend in patients with DCM, without statistical significance. These findings show the possible roles of CLICs in left ventricular myocardial remodeling under pressure overload and in DCM.

Mitochondria are key organelles responsible for cellular energy production. Excessive production of ROS causes myocardial structural remodeling and impaired cardiac function, which is significantly associated with the progression of heart failure. As heart failure progresses, the ECM undergoes dramatic changes, leading to cardiac fibrosis. One of the key insights of this study is the upregulation of *CLIC4* and *CLIC5* in TAC models and in patients with DCM. CLIC proteins, particularly CLIC4 and CLIC5, have been identified in cardiac mitochondria. CLIC4 is enriched in the outer mitochondrial membrane, whereas CLIC5 is localized in the inner mitochondrial membrane (Gururaja Rao et al., 2020; Ponnalagu et al., 2016). In addition to their canonical roles as ion channels, CLICs have been shown to influence oxidative stress and mitochondrial function, which are hallmarks of heart failure progression. For example, CLIC4 has been implicated in ROS generation and mitochondrial permeability transition pore regulation, linking it to metabolic dysfunction and apoptosis in stressed cardiomyocytes (Bayeva et al., 2013; Ponnalagu et al., 2016). Additionally, CLIC5 directly modulates mitochondrial ROS generation (Gururaja Rao et al., 2020; Ponnalagu et al., 2016). This upregulation may correlate with increased mitochondrial dysfunction, oxidative stress, and fibrosis, which are the hallmarks of heart failure. The upregulation of *CLIC1* and *CLIC4* observed in our study, combined with their strong correlations with actinins (*ACTN2* and *ACTN4*) and collagens (*COL4A1* and *COL6A2*), suggests a dual role in cytoskeletal stability and mitochondrial resilience under chronic stress. Targeting mitochondrial dysfunction in heart failure is a promising therapeutic approach that focuses on improving glucose metabolism, reducing oxidative stress, and modulating ion channels (Banović & Ristić, 2016). Mitochondrial ion channels, including those involved in chloride transport, may be potential cardioprotective targets against ischemia/reperfusion injury and heart failure progression (Hausenloy et al., 2020; Nomura et al., 2018). Our findings regarding CLICs align with previous studies that link mitochondrial dysfunction to heart failure pathophysiology through its impact on energy production, calcium handling, and ROS generation (Bayeva et al., 2013). Therefore, targeting CLICs may constitute a therapeutic strategy to prevent or reverse mitochondrial dysfunction, ECM remodeling, and heart failure progression. However, further studies are warranted to validate the potential of CLICs as therapeutic targets.

CLICs proteins are associated with the ERM proteins: ezrin, radixin and moesin. ERM proteins act as cross‐linkers between membranes and the actin cytoskeleton (Jiang et al., 2014). In the present study, pathway analysis further supported the pivotal roles of CLIC1 and CLIC4 in ECM remodeling, revealing significant enrichment in the ECM‐focal adhesion and actin cytoskeleton regulation pathways. In contrast, although *CLIC5* showed increased expression in the TAC models, its role in DCM appeared to be less critical, with no significant upregulation observed in patients with DCM. The upregulation of fibrotic markers, such as *TIMP1*, *TIMP3*, and *COL4A1*, along with CLICs, suggests their direct involvement in driving the fibrotic response in pressure‐overloaded and failing hearts. This process involves activation of myofibroblasts, inflammatory responses, and upregulation of TGF‐β signaling (Creemers & Pinto, 2011; Frangogiannis, 2019). TIMP1 promotes myocardial fibrosis through a novel mechanism involving CD63‐integrin β1 interaction, independent of its MMP‐inhibitory function (Takawale et al., 2017). ECM remodeling differs between pressure and volume overload, with distinct patterns of collagen deposition and MMP activation (Hutchinson et al., 2010). Understanding these mechanisms is crucial for developing targeted therapies to address cardiac fibrosis in various heart failure conditions (Frangogiannis, 2019). In the present study, KEGG pathway analysis identified focal adhesion and ECM‐related signaling as key pathways associated with *CLIC1*, *CLIC4*, and *CLIC5*. The consistent upregulation of *CLIC1* and *CLIC4* in both the TAC and DCM models emphasizes their primary roles in ECM remodeling. However, *CLIC5*'s role appears to be context dependent. These findings highlight the potential roles of CLIC proteins in maintaining cardiac structural integrity and mediating ECM remodeling under pressure overload. However, further in vivo studies using specific blockers of CLIC channels are required to confirm this hypothesis.

The study findings further emphasized the possible roles of CLICs, particularly CLIC1, CLIC4, and CLIC5, in cardiac hypertrophy and heart failure. Jiang et al. (2017) showed that these CLICs play an important role in the development of AF and that interaction with structural type IV collagen may promote AF development in rheumatic mitral valve disease. In the present study, the upregulation of *CLIC1* and *CLIC4* in the TAC model and patients with DCM underscores their conserved role in myocardial fibrosis. This was further supported by their associations with ECM‐related genes, such as *COL4A1*, *COL6A2*, and *CTGF*, as identified by the KEGG pathway analysis. These genes are integral to ECM remodeling, which is pivotal in pathological cardiac remodeling. The observed trend of increased *CLIC5* expression in patients with DCM suggests its potential contribution to disease pathology, although its involvement appears to be less prominent than that of *CLIC1* and *CLIC4*. The dynamic nature of ECM remodeling involves matrix metalloproteinases and their inhibitors, with alterations in their balance observed in failing hearts (Graham et al., 2008). These findings highlight the multifaceted role of ECM remodeling in heart failure progression, involving both structural and molecular changes (Li et al., 2014; Louzao‐Martinez et al., 2016). Taken together, these findings suggest that CLICs play a pivotal role in orchestrating cardiac fibrosis, ECM remodeling, and cellular adaptation in response to mechanical stress, thereby providing insights into the molecular mechanisms underlying pathological remodeling in heart failure. The similarities between the TAC model and patients with DCM, particularly the consistent upregulation of CLICs and the involvement of ECM‐related pathways, underline the clinical relevance of our findings.

This study has some limitations. First, although we utilized a well‐established TAC mouse model to simulate pressure overload‐induced cardiac remodeling, this study lacked direct functional assessments such as echocardiography or heart weight measurements. Therefore, the disease stage should be interpreted as pressure overload–induced remodeling rather than definitive heart failure, and the extrapolation of findings to human pathophysiology should be approached with caution. Second, the sample size for single‐cell RNA‐seq analysis, particularly for human DCM tissues, was limited, which may affect the generalizability of the results. Third, although our correlation and pathway analyses revealed significant associations between CLICs and ECM‐related pathways, functional validation through genetic or pharmacological manipulation of CLICs was not performed. Future studies using loss‐ or gain‐of‐function approaches and the immunohistochemical/immunofluorescent analysis of CLICs used to decipher their localization in cardiac cells will be essential to elucidate the causal role of CLICs in cardiac structural remodeling. Lastly, in the present study, we only investigated the roles of CLICs on single cardiac myocytes, and further studies are needed to clarify the pathophysiological roles of CLICs in cells other than cardiomyocytes, such as fibroblasts and endothelial cells.

## 5 CONCLUSION

In conclusion, this study using single‐cell RNA‐seq reported the possible role of CLICs in myocardial remodeling linked to ECM in the left ventricle under pressure overload and in patients with DCM, proposing their potential as therapeutic targets for cardiac hypertrophy and failure.

## AUTHOR CONTRIBUTIONS

**Gaku Oguri**: Data curation, formal analysis, visualization, writing—original draft, writing—review and editing, investigation. **Seitaro Nomura:** Data curation, formal analysis, writing—original draft, writing—review and editing, investigation. **Takafumi Nakajima**: Data curation, formal analysis, visualization, writing—original draft, writing—review and editing, investigation. **Hironobu Kikuchi:** Data curation, formal analysis, writing—review and editing, investigation. **Syotaro Obi**: Data curation, formal analysis, writing—review and editing, investigation. **Issei Komuro**: Writing—review and editing, supervision. **Norihiko Takeda**: Writing—review and editing, supervision. **Shigeru Toyoda**: Writing—review and editing, supervision. **Toshiaki Nakajima**: Data curation, formal analysis, visualization, writing—original draft, writing—review and editing, investigation.

## FUNDING INFORMATION

This study was supported in part by Japan Society for the Promotion of Science (JSPS) KAKENHI [Grant Numbers 18K15840 (Gaku Oguri) and 22H03457 (Toshiaki Nakajima)]. The funding sources had no involvement in the study design, data collection, analysis, interpretation, writing of the manuscript, or the decision to submit the article for publication.

## CONFLICT OF INTEREST STATEMENT

The authors declare that they have no known competing financial interests or personal relationships that could have appeared to influence the work reported in this paper.

## ETHICS STATEMENT

All animal experiments were approved by the Ethics Committee for Animal Experiments of the University of Tokyo (Approval number: RAC150001) and were conducted in strict accordance with animal experiment guidelines. All human experiments were approved by the Ethics Committee of the University of Tokyo (Approval number: G‐10032, approved on July 21, 2015). All procedures were conducted in accordance with the Declaration of Helsinki.

## PATIENT CONSENT

Written informed consent was obtained from all participants prior to their inclusion in the study, and the privacy rights of the study participants were observed throughout the study.

## Data Availability

Data will be made available upon reasonable request.
